# Cardiovascular Disease and Its Implication for Higher Catastrophic Health Expenditures Among Households in Sub-Saharan Africa

**DOI:** 10.36469/001c.70252

**Published:** 2023-03-17

**Authors:** Folashayo Ikenna Peter Adeniji, Taiwo Akinyode Obembe

**Affiliations:** 1 Department of Health Policy & Management, Faculty of Public Health, College of Medicine University of Ibadan, Ibadan, Nigeria

**Keywords:** cardiovascular disease, catastrophic health expenditure, universal health coverage, chronic noncommunicable diseases, sub-Saharan Africa

## Abstract

**Background:** Cardiovascular diseases (CVDs) impose an enormous and growing economic burden on households in sub-Saharan Africa (SSA). Like many chronic health conditions, CVD predisposes families to catastrophic health expenditure (CHE), especially in SSA due to the low health insurance coverage. This study assessed the impact of CVD on the risks of incurring higher CHE among households in Ghana and South Africa. **Methods:** The World Health Organization (WHO) Study on Global AGEing and Adult Health (WHO SAGE), Wave 1, implemented 2007-2010, was utilized. Following standard procedure, CHE was defined as the health expenditure above 5%, 10%, and 25% of total household expenditure. Similarly, a 40% threshold was applied to household total nonfood expenditure, also referred to as the capacity to pay. To compare the difference in mean CHE by household CVD status and the predictors of CHE, Student’s *t*-test and logistic regression were utilized. **Results:** The share of medical expenditure in total household spending was higher among households with CVD in Ghana and South Africa. Households with CVD were more likely to experience greater CHE across all the thresholds in Ghana. Households who reported having CVD were twice as likely to incur CHE at 5% threshold (odds ratio [OR], 1.946; confidence interval [CI], 0.965-1.095), 3 times as likely at 10% threshold (OR, 2.710; CI, 1.401-5.239), and 4 times more likely to experience CHE at both 25% and 40% thresholds, (OR, 3.696; CI, 0.956-14.286) and (OR, 4.107; CI, 1.908-8.841), respectively. In South Africa, households with CVD experienced higher CHE across all the thresholds examined compared with households without CVDs. However, only household CVD status, household health insurance status, and the presence of other disease conditions apart from CVD were associated with incurring CHE. Households who reported having CVD were 3 times more likely to incur CHE compared with households without CVD (OR, 3.002; CI, 1.013-8.902). **Conclusions:** Our findings suggest that CVD predisposed households to risk of higher CHE. Equity in health financing presupposes that access to health insurance should be predicated on individual health needs. Thus, targeting and prioritizing the health needs of individuals with regard to healthcare financing interventions in SSA is needed.

## BACKGROUND

Cardiovascular diseases (CVDs) are defined as a group of heart and blood vessel–related disorders.^1^ Examples of CVDs include cerebrovascular disease, hypertension, congenital heart disease, myocardial infarction, coronary artery disease or angina pectoris, rheumatic heart disease, cardiac arrhythmias, peripheral vascular disease (aneurysms and peripheral arterial disease), valvular heart disease, and congestive heart failure.^2^ These diseases impose an enormous and growing economic burden on individuals and households in developing countries. Research has shown that CVDs manifest about 10 to 15 years earlier in low- and middle-income countries (LMICs) when compared with developed countries.^3^ This earlier onset could lead to a reduction in the productive/working-age population, with serious implications for the economies of these countries.^4^

In particular, CVDs caused twice the number of deaths related to HIV, malaria, and tuberculosis combined in LMICs from the review of changes in mortality between 1990 and 2001.^5^ The Global Burden of Disease study estimated that CVDs caused almost 6.4 million deaths of people aged 30 to 69 years in developing countries in 2020.^6^ Another study estimated that 15.9 million potential productive years of life lost were lost in India and China in 2000 due to CVDs^7^ and about 2.5 billion, 237 billion, and 558 billion in 1998 International dollars lost in the gross domestic product in Tanzania, India, and China, respectively, between 2005 and 2015.^7^ As such, reducing morbidity and mortality due to CVDs remains at the center of public discourse and population health priorities in LMICs.^8^

### Epidemiology of CVD in Sub-Saharan Africa

According to the World Health Organization (WHO) Global Burden of Disease Study, CVD is one of the leading causes of morbidity and mortality in sub-Saharan Africa (SSA), with high systolic blood pressure accounting for 56.6% of disability-adjusted life-years in 2021.^9^ The number of deaths due to these diseases increased by 50% between 1990 and 2019 in SSA.^10^ In 2019 alone, CVDs caused nearly 1 million deaths in SSA, contributing about 4.5% of all CVD-related mortality globally.^11^

Although research evidence suggests few cases of CVDs were recorded in SSA before the 1990s, its prevalence has been increasing in the last 3 decades.^12^ According to studies conducted in some SSA countries, the emerging public and population health problems posed by CVDs in the subregion can be attributed to aging, rapid urbanization, demographic transition, lifestyle change, and a high prevalence of common risk factors such as hypertension, tobacco use, abdominal obesity, physical inactivity (sedentary lifestyle), alcohol misuse, and diabetes.^13^ In the INTERHEART Africa study,^13^ the 5 CVD risk factors modeled in the study were estimated to account for population attributable risk of 90% for acute myocardial infarction. In particular, current and former tobacco use, hypertension, and diabetes were revealed as the risk factors with the highest influence on the odds of a patient suffering a cardiovascular event in the Black African population. Simply put, these risk factors account for the largest percentage of the economic burden of CVD in SSA. The study suggests that the failure to control or prevent risk factors and risk behaviors will have a substantial impact on the economic burden of CVDs in Africa compared with the rest of the world in the near future.^13^

Some research evidence has shown that several other factors contribute to the risk of CVD events. For instance, higher-income Black Africans were more predisposed to myocardial infarction compared with Whites and non-Black Africans of similar economic status.^13^ The effects of the prevalence of HIV/AIDS has been linked to generating other CVDs such as rheumatic valvular and cardiomyopathy, which manifests in the form of tuberculous pericarditis.^14^

In particular, many countries in SSA are experiencing an increase in the prevalence of chronic diseases like CVD.^15^ For example, Nigeria is the most populous country in SSA, and CVDs represent a growing public health threat in the country. According to WHO estimates of the burden of noncommunicable diseases globally, CVDs were responsible for about 7% of deaths in Nigeria in 2014 alone.^16^ Similarly, several subpopulation studies have revealed that hypertensive heart failure contributes the largest burden of CVDs in Nigeria, constituting nearly 61% of all CVD cases in the country.

The Institute for Health Metrics and Evaluation reported that mortality as a result of stroke and ischemic heart disease increased by 10.6% and 14.6%, respectively, in Ghana between 2007 and 2017. In terms of the risk factors that contributed to disabilities in the same period, diabetes increased by 52.8% while the deaths and disabilities caused by high blood pressure increased by 17.0%.^17^ In South Africa, reports show that CVD is second only to HIV/AIDS in terms of the number of deaths caused. This disease condition accounts for almost 17.3% of adult deaths in the country, and more South Africans die as a result of CVDs than of all cancers.^18^

More importantly, given the weak insurance infrastructure in many SSA countries, CVDs have implications for higher catastrophic health expenditures (CHE), especially among economically less viable households. Compared with developed countries and most other developing regions of the world, SSA countries allocate significantly lower funds (as a percentage of gross domestic product) to providing healthcare services.^18^ A systematic review of public financing of healthcare services in developing countries between 1995 and 2006 revealed that while the share of government spending on health increased in most regions, it decreased in SSA countries.^19^ Also, a WHO report on health spending and disease burden in Africa indicated that healthcare remains underfunded in the region. As of 2015, only 6 SSA countries (Liberia, Madagascar, Malawi, Rwanda, Togo, and Zambia) have met the target of 15% of annual national budget set at the Abuja Declaration of 2001. (The Declaration has been used to refer to the conference held in Abuja, the federal capital territory of Nigeria, where all the members of African Union met and pledged to allocate ≥15% of the national budget to the healthcare sector yearly.) Consequently, individuals and households in SSA are often predisposed to incurring substantial CHE due to high out-of-pocket (OOP) payments for medical services.^20^ For the 2 countries used as case studies, a recent study reported that only 13.3% of the South African population had health insurance,^21^ while another study reported poor coverage of the National Health Insurance Scheme in Ghana,^22^ which means that the progress in terms of universal health coverage (UHC) has been marginal in the last decade in both countries and, indeed, in many SSA countries.

The extent of CHE experienced by individuals and households vary according to health needs (especially for chronic health conditions like CVD) and the availability of effective financial protection mechanism. Earlier studies in SSA have investigated the incidence of CHE in the general population. By extension, this study examined the differentials in the experience of CHE among households with and without CVD using data from 2 SSA countries, Ghana and South Africa. Evidence in the literature reveals that these countries are in various stages in terms of realizing UHC as articulated in the Sustainable Development Goals. Moreover, both countries have yet to provide adequate financial protection that covers the entire population.^23^ The implication is that the policy on affordable healthcare through social health insurance has yet to attain its optimal potential in both countries. Therefore, this study is warranted to examine the differentials in the experience of CHE among households with and without CVDs in these 2 countries. Findings from this study will provide evidence-based recommendations to support policies toward UHC in both countries and in other similar countries in SSA.

## METHODS

### Data Source

This study utilized data drawn from the WHO Study on Global AGEing and Adult Health (WHO SAGE), Wave 1, which was carried out between 2007 and 2010. This survey is a nationally representative study implemented in 6 countries: China, Ghana, India, Mexico, Russia, and South Africa. Only the 2 SSA countries covered in the survey were included in this study. The WHO SAGE, Wave 1 sampled 5110 and 4223 adults 50 years and older in Ghana and South Africa, respectively. Also, the study survey sampled a smaller comparative sample of adults 18 to 49 years of age. The study combined household-level and individual-level modules to collect important data sets. The household-level module was used to elicit information such as household healthcare demand and utilization, access to medical insurance, broad categories of household spending, including health expenditure, and household permanent income. The expenditure categories elicited in the data include monthly expenditure on food items; housing and utility; clothing, transportation, recreation, and entertainment; and total monthly healthcare spending. Expenditures on education, durable goods, vehicles, cost of hospitalization, and amount paid on insurance premiums were elicited on an annual basis. Similarly, the individual-level module was used to collect data on variables such as sociodemographics, health risk behavior, and health state description. In the data set, household health expenditure aggregated the monetary outlay toward the outpatient care received from physicians and nurses, purchase of medicines/drugs, diagnostic and laboratory tests, traditional or alternative care, costs associated with hospitalizations, and other medical-related costs. The health conditions covered in the data included arthritis, stroke, angina or angina pectoris, chronic lung cancer, asthma, depression, hypertension, cataracts, oral health, injuries, and cervical and breast cancer (for women only). Based on the standard classification of diseases, angina and hypertension are the 2 CVD conditions captured in the data. Therefore, these 2 heart-related conditions were utilized as CVDs for the purpose of data analysis.

### Measure of Catastrophic Health Expenditure

The conceptualization of CHE relates to medical spending above a predetermined threshold, in relation to household income, that often causes financial distress for individuals and households. This study favored the use of household expenditure as a measure of household income and thus as a proxy for household economic well-being. This is because consumption expenditure is less affected by short-term fluctuations and better reflects the welfare of households.^24^ Therefore, in this study, CHE is defined as the medical expenditure above 5%, 10%, and 25% of household total expenditure. Similarly, a 40% threshold was applied to household total nonfood expenditure, which has also been widely utilized in the literature. Household expenditure categories were adjusted for household composition and size. Thus, the level of CHE among households was implemented as follows:

$$CHE=\frac{He}{heor(he-hfex)}\geq z\%$$

where *He* = total household health expenditure, *he* = total household expenditure, *hfex* = total household food expenditure, *he−hfex* = capacity to pay/discretionary income, and *z*% = the predetermined thresholds (which are 5%, 10%, 25%, and 40%, depending on the denominator). To adjust for household size, all expenditure categories were divided by the respective household size to generate per capita expenditure categories. The share of health expenditure in household budget was expressed as:

$$\left(\frac{\text{Total Household Health Expenditure}(H_{e})}{\text{Total Household Expenditure}(h_{e})}\right)\times 100$$

### Data Analysis

For comparison purposes, households were classified by their CVD status (ie, whether a member or members of the household reported having CVD. The data from both countries were analyzed independently, and the results were compared accordingly. Descriptive statistics were utilized to report categorical variables, and the mean and SD of continuous variables were presented. To measure the CHE among households, the standard procedures adopted in previous studies was used.^25,26^ All expenditure categories were annualized. To compare the difference in mean CHE by household CVD status and the predictors of CHE, Student’s *t*-test and logistic regression were utilized. The logistic regression model is given as:

$$\frac{\pi }{1-\pi }=exp\left(\beta_{0}+\beta_{1}X_{1}+\ldots +\beta_{k}X_{k}\right),$$

where π ∕ 1 − π is the probability for the event of CHE (ie, the outcome variable) and *X*_1_ represents the explanatory/predictor variables. Important risk factors for CVD were identified from literature reviews and were included in the logistic regression model as *X*_1_. All predictors were included and retained. A sensitivity analysis, using different thresholds of CHE, was then used to determine what variables are significant at different thresholds. All the data were analyzed using Stata version 15 (StataCorp, College Station, Texas).

### Predictor Variables

The covariates controlled for in the model include sex, age, and education of household head; tobacco use; alcohol use; CVD status; household access to health insurance; number of fruit servings; number of vegetable servings; daily vigorous physical activity; household per capita expenditure; household size; and presence of other non-CVD health conditions such as the presence of cervical and breast cancers (for women), arthritis, stroke, chronic lung disease, asthma, depression, cataracts, oral health, and injuries. A dummy variable was introduced in the regression model such that 1 (Yes) represented the presence of 1 or more of the non-CVD health conditions captured in the data; and 0 (No) for households who did not report any of the non-CVD conditions. The number of fruits and vegetable servings were captured per day and per year, while vigorous physical activities were captured per day and per week. Weekly and monthly data were annualized for data analysis. Covariates such as sociodemographic characteristics, tobacco use, alcohol use, number of fruit servings, number of vegetable servings, and daily vigorous physical activity were included in the model because they have been strongly associated with increasing or reducing the risks of CVDs and other chronic noncommunicable diseases apart from other nonmodifiable risk factors like age and family history.^27,28^ The intuition is that when an individual engages in a risky health behavior and falls ill as a result, there is a tendency that the individual could incur CHE due to increased demand for healthcare services. Although indirect, the path from risky health behavior to CHE affects the demand for healthcare services.

## RESULTS

**Table 1** depicts the sociodemographic characteristics of households by CVD status in Ghana and South Africa. Data on 3938 individuals sampled in Ghana and 2400 persons sampled in South Africa were analyzed. In both countries, the mean age in households with CVD (Ghana, 49.28 ± 17.84 years; South Africa, 48.96 ± 15.85 years) was higher compared with households without CVD (Ghana, 40.66 ± 16.46 years; South Africa, 35.90 ± 13.25 years). There were more males relative to females who had CVD (147 [74.62] in Ghana and 187 [67.03%] in South Africa). These figures also indicate that the prevalence of CVD is relatively higher in the latter country. Similarly, a higher proportion of respondents who were currently married had CVD in both countries, 128 (64.97%) and 142 (50.90%), respectively. The level of access to health insurance reported among households was generally low. Among households without CVD, the majority (96.87% and 88.87% in both countries, respectively) reported not having access to health insurance. Likewise, in Ghana, 95.83% of households with member(s) with CVD had no access to financial protection in the form of health insurance. Also, in South Africa, only 10.66% of households with CVD reported having access to health insurance. Access to health insurance is slightly higher in South Africa than in Ghana.

**Table 1.** Sociodemographic Variables by CVD Status in Ghana and South Africa (WHO SAGE, Wave 1)

**Variable**	**Ghana, n (%)**			**South Africa, n (%)**		
	**No CVD**	**CVD**	**Total**	**No CVD**	**CVD**	**Total**
Age (y)	40.66 (45.21)	49.28 (54.79)	44.97^a^ (100.00)	35.90 (42.32)	48.96 (57.70)	42.43^a^ (100.00)
Education (y)	6.10 (54.61)	5.07 (45.39)	6.03^a^ (100.00)	8.95 (5.57)	6.84 (6.01)	8.65^a^ (100.00)
Sex						
Male	1468 (53.25)	147 (74.62)	1615 (54.61)	1001 (50.89)	187 (67.03)	1236 (52.57)
Female	1289 (46.75)	50 (25.38)	1339 (45.39)	966 (49.11)	92 (32.97)	1115 (47.43)
Marital status						
Never married	576 (20.92)	20 (10.15)	596 (20.31)	972 (49.49)	67 (24.01)	1094 (46.59)
Currently married	1618 (58.75)	128 (64.97)	1746 (59.07)	723 (36.81)	142 (50.90)	899 (38.29)
Cohabiting	74 (2.69)	5 (2.54)	79 (2.67)	27 (1.37)	2 (0.72)	31 (1.32)
Separated/divorced	196 (7.12)	15 (7.61)	211 (7.18)	56 (2.85)	14 (5.02)	71 (3.02)
Widowed	249 (9.03)	29 (14.72)	278 (9.36)	93 (4.74)	43 (15.41)	144 (6.13)
Unknown	41 (1.49)	—	41 (1.40)	93 (4.74)	11 (3.94)	109 (4.64)
Health insurance						
No	1947 (96.87)	138 (95.83)	2205 (96.50)	1621 (88.87)	243 (89.34)	2192 (89.51)
Yes	63 (3.13)	6 (4.17)	69 (3.50)	203 (11.13)	29 (10.66)	257 (10.49)

Age, education, sex, and marital status are strictly that of the household head. Abbreviation: CVD, cardiovascular disease.

^a^Mean age and education of the total sample, in years.

Tobacco use, alcohol misuse, low fruit and vegetable intake, and inadequate physical activity are among the major risk factors for CVD. **Table 2** shows the summary of common risk factors for CVD among households in the 2 SSA countries. The prevalence of tobacco use in South Africa (73.84%) is very high compared with Ghana (7.48%). However, more households reported alcohol consumption in Ghana (45.31%) than South Africa (29.77%). Similarly, the percentage of households that consumed tobacco and reported having CVD in Ghana (6.34%) is lower relative to that of South Africa (14.28%). The mean fruit and vegetable servings among households with CVD (4.02 and 3.06) is higher than that among households without CVD (3.90 and 2.51). In South Africa, the average number of fruit servings among households with CVD (1.96) is also higher relative to households without CVD (1.88). In both countries, households without CVDs had greater rigorous physical activity on average.

**Table 2.** Risk Factors for CVD in Ghana and South Africa (WHO SAGE, Wave 1)

**Risk Factor**		**Ghana**			**South Africa**	
	**No CVD**	**CVD**	**Total**	**No CVD**	**CVD**	**Total**
Tobacco use, n (%)						
No	2535 (92.52)	184 (93.4)	2752 (92.6)	515 (26.3)	73 (26.16)	627 (26.9)
Yes	205 (7.48)	13 (6.6)	220 (7.4)	1443 (73.7)	206 (73.84)	1704 (73.1)
Alcohol use, n (%)						
No	1499 (54.69)	106 (53.81)	1626 (54.69)	1373 (70.23)	192 (68.82)	1628 (69.96)
Yes	1242 (45.31)	91 (46.19)	1347 (45.31)	582 (29.77)	87 (31.18)	699 (30.04)
No. of fruit servings, mean (SD); range	3.90 (4.01); 0.00-50.00	4.02 (4.36); 0.00-40.00	3.91 (4.08); 0.00-50.00	1.88 (1.57) 0.00-12.00	1.96 (1.98); 0.0-21.00	1.87 (1.63); 0.00, 21.00
No. of vegetable servings, mean (SD); range	2.51 (1.92); 0.00-50.00	3.06 (3.91); 0.00-50.00	2.55 (2.11); 0.00-50.00	1.96 (1.53); 0.00-12.00	1.95 (1.53); 0.00-12.00	1.96 (1.61); 0.00-21.00
Vigorous physical activities, mean (SD); range	2.510 (2.700); 0-7	1.363 (2.310); 0-7	2.431 (2.691); 0-7	1.099 (1.940); 0-7	0.743 (1.604); 0-7	1.036 (1.891); 0-7

Abbreviation: CVD, cardiovascular disease.

The mean share of health expenditure in relation to household expenditure by CVD status is shown in **Table 3**. This share was calculated using total household expenditure and total household nonfood expenditure or capacity to pay, respectively. The share of medical expenditure in household expenditure and nonfood expenditure was higher among with CVD patients in Ghana. Likewise, in South Africa, the share of health spending in total expenditure was similar across households irrespective of whether the household had CVD. Nonetheless, the share of medical expenditure with respect to total household nonfood expenditure was higher among households with CVD in South Africa.

**Table 3.** Health Expenditure Share of Total Household Expenditure in Ghana and South Africa (WHO SAGE, Wave 1)

		**Ghana**			**South Africa**	
	**No CVD (n = 2098)**	**CVD** **(n = 158)**	**Total** **(n = 2394)**	**No CVD (n = 1325)**	**CVD** **(n = 192)**	**Total** **(n = 1775)**
Share of total household expenditure, mean (SD); range	0.04 (0.06); 0.00, 0.63	0.06 (0.09); 0.00, 0.49	0.040.07; (0.00, 0.87)	0.02 (0.06); 0.00, 0.78	0.02 (0.07); 0.00, 0.47	0.02 (0.06); 0.00, 0.78
Share of total household nonfood expenditure, mean (SD); range	0.11 (0.15); 0.00, 0.89	0.17 (0.22); 0.00, 0.95	0.12 (0.16); 0.00, 0.96	0.03 (0.10); 0.00, 0.94	0.04 (0.12); 0.00, 0.78	0.03 (0.10); 0.00, 0.94

Abbreviation: CVD, cardiovascular disease.

**Table 4** shows the CHE head count by household CVD status in the 2 countries. The level of CHE among households in both countries was higher with the 5% threshold compared with the 10%, 25%, and 40% thresholds. In Ghana and at the 5% threshold, the level of CHE was 27.78% among households without CVD relative to 37.34% among households with CVD. At the 10% threshold, more households with CVDs (24.05%) incurred CHE relative to those without CVD (12.39%) in Ghana. This trend was consistent for other thresholds. Also in South Africa, households that reported having a member with CVD experienced higher CHE across all the thresholds examined compared with households without CVD.

**Table 4.** Catastrophic Health Expenditures by CVD Status Among Households (WHO SAGE, Wave 1)

**CHE Threshold Level**	**Ghana**			**South Africa**		
	**No CVD**	**CVD**	**Total**	**No CVD**	**CVD**	**Total**
5%, n (%)						
No	1516 (72.22)	99 (62.66)	1615 (71.56)	1202 (90.72)	172 (89.58)	1374 (90.57)
Yes	583 (27.78)	59 (37.34)	642 (28.44)	123 (9.28)	20 (10.42)	143 (9.43)
10%, n (%)						
No	1839 (87.61)	120 (75.95)	1959 (86.80)	1257 (94.87)	180 (93.75)	1437 (94.73)
Yes	260 (12.39)	38 (24.05)	298 (13.20)	68 (5.13)	12 (6.25)	80 (5.27)
25%, n (%)						
No	2057 (98.00)	150 (94.94)	2207 (97.78)	1304 (98.42)	186 (96.88)	1490 (98.22)
Yes	42 (2.00)	8 (5.06)	50 (2.22)	21 (1.58)	6 (3.13)	27 (1.78)
40%, n (%)						
No	1933 (92.09)	135 (85.44)	2068 (91.63)	1297 (97.89)	186 (96.88)	1483 (97.76)
Yes	166 (7.91)	23 (14.56)	189 (8.37)	28 (2.11)	6 (3.13)	34 (2.24)

Abbreviations: CHE, catastrophic health expenditure; CVD, cardiovascular disease.

The difference in mean CHE is compared among households by CVD status as shown in **Table 5**. In Ghana, estimates show that households who reported having CVD significantly incurred CHE compared with households without CVD for all the thresholds. There is a similar pattern for households in South Africa, although it was significant only for the 25% threshold. For the 5%, 10%, 25%, and 40% thresholds, households with CVD incurred about 9%, 11%, 3%, and 6% higher CHE, respectively, relative to the comparison group in Ghana. However, in South Africa, this difference is less evident, except for the 25% threshold, where households with CVD incurred roughly 2% higher CHE compared with households without CHE.

**Table 5.** Student’s *t*-Test Results Comparing the Difference in Mean CHE by CVD Status (WHO SAGE, Wave 1)

**CHE Threshold Level**	**Ghana**		**South Africa**	
	**Estimated Difference (SE)**	**95% CI**	**Estimated Difference (SE)**	**95% CI**
5%	-0.096^a^ (0.037)	-0.168, -0.023	-0.011 (0.023)	-0.056, 0.033
10%	-0.1166^a^ (0.028)	-0.171, -0.062	-0.011 (0.017)	-0.045, 0.023
25%	-0.031^a^ (0.012)	-0.054, -0.007	-0.015^b^ (0.010)	-0.035, 0.005
40%	-0.066^a^ (0.022)	-0.111, -0.02	-0.010 (0.011)	-0.033, 0.012

Abbreviations: CHE, catastrophic health expenditure; CI, confidence interval; CVD, cardiovascular disease; SE, standard error.

^a^Significant at *P* < .01.

^b^Significant at *P* < .1.

**Table 6** depicts the factors associated with the experience of CHE among households with and without CVD. In Ghana, sex, age, CVD status, engagement in daily vigorous activity, household per capita expenditure, and household size were associated with CHE across all the thresholds. For instance, households with CVD were more likely to experience greater CHE across all the thresholds examined in Ghana. Households who reported having CVD were twice as likely to incur CHE at the 5% threshold (OR, 1.946; CI, 0.965-1.095), 3 times more likely at 10% threshold (OR, 2.710; CI, 1.401-5.239), and 4 times more likely to experience CHE at both 25% and 40% thresholds (OR, 3.696; CI, 0.956-14.286), and (OR, 4.107; CI, 1.908-8.841), respectively. In South Africa, only household CVD status, household health insurance status, and the presence of other disease conditions apart from CVD were associated with incurring CHE. Households who reported having CVD were 3 times more likely to incur CHE than households without CVD (OR, 3.002; CI, 1.013-8.902).

**Table 6.** Factors Associated With CHE Among Households With and Without CVD in Ghana and South Africa (WHO SAGE, Wave 1)

	**CHE Threshold Level: Ghana**								**CHE Threshold Level: South Africa**							
	**5%**		**10%**		**25%**		**40%**		**5%**		**10%**		**25%**		**40%**	
	**OR (SE)**	**95% CI**	**OR (SE)**	**95% CI**	**OR (SE)**	**95% CI**	**OR (SE)**	**95% CI**	**OR (SE)**	**95% CI**	**OR (SE)**	**95% CI**	**OR (SE)**	**95% CI**	**OR (SE)**	**95% CI**
Sex	1.070 (0.168)	0.786-1.457	1.399 (0.305)	0.912-2.145	2.998^b^ (1.610)	1.046-8.589	2.274^c^ (0.596)	1.361-3.801	0.998 (0.211)	0.660-1.509	0.985 (0.274)	0.571-1.697	1.842 (0.849)	0.747-4.544	1.397 (0.563)	0.634-3.078
Age (y)	1.742^c^ (0.352)	1.172-2.590	1.321 (0.354)	0.781-2.233	0.792 (0.449)	0.261-2.406	0.895 (0.303)	0.461-1.739	0.814 (0.219)	0.480-1.381	1.185 (0.385)	0.627-2.242	0.951 (0.509)	0.333-2.715	1.339 (0.608)	0.550-3.261
Education (y)	0.908 (0.136)	0.678-1.217	1.187 (0.245)	0.792-1.779	1.233 (0.656)	0.434-3.500	1.017 (0.255)	0.622-1.663	0.700^a^ (0.130)	0.486-1.008	0.677^a^ (0.144)	0.447-1.026	1.206 (0.449)	0.581-2.504	1.156 (0.354)	0.634-2.105
Tobacco use	1.072 (0.086)	0.917-1.254	1.030 (0.108)	0.838-1.266	1.197 (0.353)	0.671-2.135	1.307 (0.253)	0.894-1.910	0.895 (0.228)	0.543-1.476	0.699 (0.233)	0.364-1.344	0.377 (0.267)	0.094-1.511	0.543 (0.283)	0.195-1.510
Alcohol use	1.018 (0.038)	0.946-1.095	0.984 (0.051)	0.889-1.090	0.945 (0.113)	0.956-14.286	1.025 (0.066)	0.904-1.162	1.054 (0.068)	0.930-1.195	1.033 (0.086)	0.878-1.215	1.178 (0.194)	0.853-1.626	1.133 (0.145)	0.882-1.456
CVD status	1.946^b^ (0.548)	1.121-3.380	2.710^c^ (0.912)	1.401-5.239	3.696^a^ (2.550)	0.956-14.286	4.107^c^ (1.607)	1.908-8.841	1.354 (0.412)	0.746-2.460	1.446 (0.524)	0.710-2.944	3.002^b^ (1.665)	1.013-8.902	1.889 (0.993)	0.674-5.294
Insurance	0.506 (0.348)	0.131-1.949	0.891 (0.711)	0.187-4.254			0.709 (0.776)	0.083-6.056	1.736^b^ (0.447)	1.048-2.877	1.699^a^ (0.545)	0.906-3.186	2.864^b^ (1.385)	1.110-7.391	2.906^b^ (1.208)	1.287-6.565
No. of fruit servings	0.996 (0.015)	0.966-1.026	1.017 (0.019)	0.980-1.054	1.033 (0.039)	0.960-1.112	1.019 (0.023)	0.976-1.065	1.036 (0.065)	0.917-1.171	1.068 (0.085)	0.914-1.249	1.006 (0.169)	0.723-1.399	0.987 (0.134)	0.755-1.289
No. of vegetable servings	1.009 (0.042)	0.930-1.095	0.966 (0.052)	0.868-1.074	0.912 (0.108)	0.723-1.150	0.928 (0.062)	0.814-1.057	0.975 (0.064)	0.856-1.109	0.996 (0.080)	0.850-1.167	0.771 (0.136)	0.545-1.090	0.933 (0.120)	0.725-1.201
Daily vigorous physical activity	1.056^a^ (0.030)	1.000-1.116	1.031 (0.038)	0.959-1.108	0.967 (0.075)	0.831-1.125	0.949 (0.042)	0.870-1.034	0.968 (0.053)	0.869-1.077	0.944 (0.074)	0.810-1.100	1.135 (0.129)	0.908-1.419	1.108 (0.108)	0.915-1.341
Household per capita expenditure	1.000^c^ (0.000)	1.000-1.000	1.000^a^ (0.000)	1.000-1.000	1.000 (0.000)	1.000-1.000	1.000^b^ (0.000)	1.000-1.000	1.000 (0.000)	1.000-1.000	1.000 (0.000)	1.000-1.000	1.000 (0.000)	0.999-1.000	1.000 (0.000)	0.999-1.000
Household size	0.936^b^ (0.028)	0.883-0.991	0.946 (0.036)	0.877-1.020	0.926 (0.068)	0.802-1.068	0.921^a^ (0.043)	0.841-1.010	0.986 (0.048)	0.896-1.086	1.015 (0.067)	0.892-1.155	1.022 (0.108)	0.831-1.257	1.070 (0.087)	0.913-1.255
Other health conditions	0.999 (0.218)	0.652-1.531	0.862 (0.264)	0.473-1.570	1.736 (1.048)	0.532-5.669	0.629 (0.284)	0.260-1.523	0.381 (0.467)	0.035-4.206	0.059^a^ (0.093)	0.003-1.296	0.004^b^ (0.011)	0.000-0.825	0.002^c^ (0.004)	0.000-0.163
Constant	0.061^c^ (0.058)	0.009-0.393	0.029^c^ (0.038)	0.002-0.391	0.003^a^ (0.010)	0.000-1.064	0.020^a^ (0.040)	0.000-1.058	0.998 (0.211)	0.660-1.509	0.985 (0.274)	0.571-1.697	1.842 (0.849)	0.747-4.544	1.397 (0.563)	0.634-3.078

The variance-covariance matrix for the fitted logistic regression was used to derive standard errors and confidence intervals of odds ratios. The reported odds ratios were unadjusted.

Abbreviations: CHE, catastrophic health expenditure; CI, confidence interval; CVD, cardiovascular disease; OR, odds ratio; SE, standard error.

^a^*P* < .01.

^b^*P* < .05.

^c^*P* < .1.

## DISCUSSION

Many chronic health conditions like CVD predispose individuals and families to CHE, especially in SSA, where the majority of the countries are still grappling with how to achieve UHC. This study assessed the spillover effects of having CVD on the risks of incurring higher CHE among households in 2 SSA countries, Ghana and South Africa. As such, this study is one of the few in SSA that examined the economic burden of CVD in this way.

As depicted in the sociodemographic background to this study, the average age of the head of households with CVD was higher than that of households without CVD. This finding is supported by previous studies where the incidence of CVD, like other chronic diseases, is reported to increase with age.^29^ Furthermore, evidence in this study revealed that the number of households covered by health insurance is low in both countries but slightly higher in South Africa than in Ghana. The implication is that there is an overreliance on OOP payments for medical care in the 2 countries, which may result in enormous financial hardship for individuals and households. A study reviewing the challenges of mobilizing substantial financial resources to achieve UHC in SSA showed that OOP spending remains the largest source of finance for healthcare services in the region, accounting for approximately 36% of current medical expenditure, which is very high relative to the global average of 22%.^30^

Likewise, the low access to health insurance reported among households in this study is reflected in the share of household medical spending in total expenditure. In Ghana, households on average spent as much as 12% of total nonfood expenditure on healthcare services. However, in South Africa, the share of medical expenditure in total nonfood spending was only 3%. More importantly, the share of medical expenditure in total household expenditure was revealed to be higher among households with CVD in both countries and across all the considered thresholds, relative to households without CVD. This finding is intuitive since chronic diseases like CVD require regular demand for healthcare services; consequently, this will lead to a larger portion of household resources devoted to accessing medical services. Earlier studies have also published findings that corroborate this evidence. In 2007, a study applied rigorous econometric modeling to investigate the economic burden of CVD in the United States. The study found that almost 30% of all medical spending among major health insurers was due to claims related to CVD treatment.^31^ Another study in the same country examined the disproportional health spending associated with various health conditions among the elderly. The study reported that the average medical outlay of older persons with chronic health conditions was 5 times greater than those without chronic disease.^32^ In particular, the study showed that expenditure for treating CVD was the highest among all chronic disease conditions. In SSA, a study on the pattern of chronic disease conditions and the self-reported financial situation of the elderly in South Africa revealed that having chronic disease was associated with household economic conditions.^33^

Moreover, the risk of incurring CHE increases with a higher economic burden of health expenditure due to increase in the demand for medical services, which is often triggered by the presence of chronic disease conditions in families. This can be seen in the level of CHE experienced among households with regard to their CVD status in this study. Evidence in Ghana and South Africa showed that the extent of CHE incurred in households with CVD was higher compared with that incurred in households without this health condition. Comparing results in both countries, CHE head count was disproportionately higher in Ghana relative to South Africa and this can be as a result of the higher access to health insurance in South Africa as revealed earlier. This suggests that Ghana and South Africa as well as other similar SSA countries need to do more to financially protect their populations to forestall the catastrophic impacts of health expenditure in the event of unanticipated health disruptions. In addition, the result in this study revealed that the difference in CHE incurred among households with a member(s) with CVD and households without persons suffering from CVD, was significantly different as shown in the *t*-test result. In the model fitted for CHE, the presence of CVD was associated with whether a household experienced CVD in Ghana across the 4 thresholds considered, whereas the effect of CVD was found significant only at the 25% threshold in South Africa. Similarly, this finding is consistent with the evidence reported in previous studies. For instance, a research conducted in China to evaluate the inequality in the experience of CHE induced by having a household member(s) with hypertension revealed that such households incurred a disconcerting level of CHE compared with families with no chronic conditions.^34^ In 2019, Haakenstad et al^35^ implemented a cross-country analysis to examine the disease-specific differentials in the incidence of CHE in many countries of the world. Their results showed that heart-related health conditions were associated with about a 1.9% increase in CHE and that this link was stronger compared with other diseases assessed. Overall, much more needs to be done to protect households from the impoverishing effects of excessive health expenditure in both countries and in other similar countries in SSA.

### Limitations

Because the majority of SSA countries are data poor, this study was limited by data availability. First, the survey utilized for this study was conducted just over 10 years ago, although a preliminary literature search showed that progress has been slow, as evidenced in the persistently low coverage of health insurance in many countries in SSA. However, the data set remains the most comprehensive and reliable source of data for this type of analysis to date. The data used for this study allowed the study to provide evidence-based information that will be useful in the design of healthcare financing policies among the countries in SSA. Nonetheless, future studies will benefit from regular surveillance of the disease-specific burden of OOP health expenditure among households in SSA. These limitations should be considered when interpreting the findings presented in this study.

## CONCLUSION

Research evidence abounds on the increase in the prevalence of CVDs in SSA, imposing substantial economic burdens on individuals and households in the region. One way to evaluate the economic burden of diseases at a microeconomic level is to assess the risk of CHE associated with the disease condition. This study found evidence that suggests that CVD predisposes households to the risk of higher CHE in SSA. Equity in health financing presupposes that access to health insurance should be predicated on individual health needs. Therefore, findings in this study emphasize the need to target and prioritize the health needs of individuals with regard to healthcare financing interventions. Furthermore, governments in Ghana and South Africa, including other countries in SSA, should intensify efforts to fully adopt and effectively implement the WHO’s Global Action Plan for the Prevention and Control of Non-Communicable Diseases. This will certainly facilitate the prevention and control of CHE related to CVDs in the region. Some of the “best buys” policy interventions recommended for curtailing chronic noncommunicable diseases include the control of tobacco use, excessive/unhealthy alcohol intake, reduction in the consumption of sugar-sweetened beverages, and health education to prevent sedentary lifestyle, among other recommendations.^36–38^ In terms of the consumption of tobacco, excessive alcohol intake and the consumption of sugar-sweetened beverages, governments can adopt highly effective tools such as regular increase in the excise taxes levied on those commodities in addition to the use of other economic and legislative tools.^36^

### Author Contributions

F.I.P.A. and T.A.O. participated in the conception of the study. F.I.P.A. wrote the first draft of the manuscript, and it was reviewed and corrected by T.A.O. Both authors approved the final version of the manuscript.

### Disclosures

The authors report no potential conflicts of interest.

### Ethics Approval and Consent

This paper used data from the WHO Study on Global AGEing and Adult Health (SAGE) implemented by the World Health Organization ICF in conjunction with the participating countries. Ethical approval for the study was obtained from the World Health Organization’s Ethical Review Board and from each site’s respective ethical board.

### Data Availability

The data set used for this study is available based on request through the WHO Multi-Country Studies Data Archive (https://apps.who.int/healthinfo/systems/surveydata/index.php/catalog).
